# Relationship between proprioception and balance control among Chinese senior older adults

**DOI:** 10.3389/fphys.2022.1078087

**Published:** 2022-12-15

**Authors:** Qi Wang, Haitao Fu

**Affiliations:** ^1^ College of Graduate Education, Shandong Sport University, Jinan, China; ^2^ Athletic Training Division, Shandong Sport University, Jinan, China

**Keywords:** balance control, tactile sensation, proprioception, muscle strength, older adult

## Abstract

**Background:** Balance impairment is the most common risk factor for falls among older adults, with three potential factors (tactile sensation, proprioception, and muscle strength) being responsible for their balance control. However, controversies remain on whether or not balance control is related to the three contributors among older adults. Therefore, clarifying the above questions helps explain the mechanisms of increased falls among senior older adults. This study compares the balance control and the three factors and investigates their relationships among older adults of different ages.

**Methods:** 166 participants ultimately passed the qualification assessment and were categorized into younger (YG, 60–69 years, *n* = 56), middle (MG, 70–79 years, *n* = 57), or older (OG, ≥80 years, *n* = 53) aged groups. Berg Balance Scale (BBS) performance, tactile sensation, proprioception, and muscle strength were tested. One-way ANOVA and partial correlation were performed to explore the differences between groups in BBS and its three potential contributors, along with the correlations between them within each age group.

**Results:** Significant differences among the three groups were detected in BBS scores (*p* < 0.001), tactile sensation at the great toe (*p* = 0.015) and heel (*p* = 0.025), proprioception of knee flexion (*p* < 0.001) and extension (*p* < 0.001), and ankle plantarflexion (*p* < 0.001) and dorsiflexion (*p* < 0.001), and muscle strength of ankle plantarflexion (*p* < 0.001) and dorsiflexion (*p* < 0.001), and hip abduction (*p* < 0.001). Proprioception of knee flexion (*r* = −0.351, *p* = 0.009) and extension (*r* = −0.276, *p* = 0.041), and ankle plantarflexion (*r* = −0.283, *p* = 0.036), and muscle strength of ankle plantarflexion (*r* = 0.326, *p* = 0.015) and hip abduction (*r* = 0.415, *p* = 0.002) were correlated with BBS among the YG. Proprioception of ankle plantarflexion (*r* = -0.291, *p* = 0.030) and muscle strength of ankle plantarflexion (*r* = 0.448, *p* = 0.001) and dorsiflexion (*r* = 0.356, *p* = 0.007) were correlated with BBS among the MG. Muscle strength of ankle plantarflexion (*r* = 0.276, *p* = 0.039) and hip abduction (*r* = 0.324, *p* = 0.015) were correlated with BBS among the OG.

**Conclusion:** YG and MG had better balance control, tactile sensation, proprioception, and muscle strength compared to OG. Proprioception correlated with balance control in YG and MG, but not in the OG. The worsen proprioception among the OG could be the key for increased falls. Exercise should be recommended to improve proprioception among senior older adults.

## 1 Introduction

Falling is a major health concern for older adults and is one of the leading causes of injury and accidental death among older adults (Kannus et al., 2005; Pizzigalli et al., 2011). One study reported that accidental falls kill more than 10,000 people aged 65 and older yearly (Shankar et al., 2017) and this risk increases with age (Scuffham et al., 2003). Over 30% of adults aged over 60 years fall at least once a year (Moreland et al., 2003; Gerards et al., 2017) with the number increasing to 60% among those aged over 80 (Gschwind et al., 2013).

Impaired balance is one of the most common risk factors leading to falling (Muir et al., 2010). Therefore, performance-based functional balance tests have been widely adopted in laboratory tests to objectively quantify said risk. Specifically, the Berg Balance Scale (BBS) is commonly used to evaluate the balance control ability of older adults, thus helping predict the risk of falling.

Balance control is essential for performing most daily life activities and involves integrating information from the musculoskeletal systems and sensory inputs (Muir et al., 2010). Tactile sensation, proprioception, and muscle strength are three potentially responsible factors for balance control among older adults (Song et al., 2021). Tactile sensation plays an essential role in balance control, while cutaneous mechanoreceptors in the foot soles provide necessary feedback for the balance control system to maintain balance (Shaffer and Harrison, 2007). Proprioception is the internal sense of body position, and is also essential for regulating balance and generating and maintaining precise movement patterns or gaits (Henry and Baudry, 2019). Motion perception is important in maintaining balance among older adults: when a disturbance occurs, the lower extremity angles (such as the ankle angles) changes dramatically. Precepting the angle changes earlier would make it easier to resist the disturbance (Song et al., 2021). As the primary support joints of the human lower extremity, proprioception input around the knee and ankle provide a component for the establishment and maintenance of functional joint stability (Lephart et al., 1998). Muscle strength is then essential to maintain an upright posture and physical balance (Gouveia É et al., 2020). When individuals are subjected to external disturbances, they first restore their bodies to its original position by applying ankle or hip torques (Horak, 2006). Ankle plantarflexion and dorsiflexion and hip abduction muscle strength are particularly critical in preventing anterior-posterior and medial-lateral falls among older adults (LaRoche et al., 2010; Arvin et al., 2016).

Previous studies have long established the existence of age-related declines in balance control (Thomas et al., 2019), tactile sensation (Shaffer and Harrison, 2007), proprioception (Henry and Baudry, 2019), and muscle strength (Doherty, 2001). The decline of balance (Muir et al., 2010), sensation (Chen and Qu, 2019), and muscle strength (Yang et al., 2018) among older adults increases their risk of falling, yet few studies have investigated whether these factors continue to decline with age in older adults at different ages. Additionally, the relationship between tactile sensation (Menz et al., 2005; Ünver and Akbaş, 2018), proprioception (Amin and Herrington, 2014; Chen and Qu, 2019), and muscle strength (Muehlbauer et al., 2012; Gouveia É et al., 2020) with balance control remains controversial. These controversies may be due to the different relationships between balance control and potential factors in various age groups. For instance, Ünver and Akbaş. (2018) demonstrated no correlation between tactile sensation and balance among older adults with a mean age of 73, while Menz et al. (2005) showed that tactile sensation was related to balance among older adults with an average age of 80 years. To our knowledge, studies are yet to explore the relationship between balance control and its potential factors among older adults in different age groups.

Therefore, this study pioneeringly compares balance control, tactile sensation, proprioception, and muscle strength across three different age groups and examines their age-specific relationships to determine the causes of increased falls among older adults. Our primary hypothesis was significant differences in balance control, tactile sensation, proprioceptive, and muscle strength among older adults of different ages. The secondary hypothesis posits that balance control was significantly correlated with tactile sensation, proprioceptive, and muscle strength among older adults from different age group.

## 2 Material and methods

### 2.1 Participants

Participants were recruited through awareness talks and the distribution of flyers in the local community. The inclusion criteria were as follows: 1) ≥ 60 years; and 2) walking independently without outside help. Meanwhile, the exclusion criteria were the following: 1) visual defects, vestibular disorders, central nervous system dysfunction, and psychological disorders associated with previous falls; 2) cognitive impairment detected through laboratory examination; and 3) self-reported trauma or disease that might significantly affect BBS, tactile sensation, proprioception and muscle strength testing such as chronic pain, history of fracture during the last 6 months, foot ulcers, osteoarthritis, diabetes, lower extremity joints prostheses and lumbar disc herniation. Following the qualification assessment, a total of 166 participants were enrolled and included in the final analysis. The age groups of the older adults were identified as younger- (YG, 60–69 years, *n* = 56), middle- (MG, 70–79 years, *n* = 57), or older- (OG, ≥80 years, *n* = 53) age groups. All participants voluntarily signed informed consent forms before the start of the test. All protocols and procedures herein were approved by the Institutional Review Boards in Shandong Sport University (19003) and followed the Helsinki Declaration.

### 2.2 BBS test

The BBS test was used for the functional assessment of balance control, which included 14 tests of simple daily functional activities such as standing with eyes closed, single leg stance, and sit-to-stand conversion (ICC value, 0.98–0.99) (Berg et al., 1992). The participants’ performance in each of these activities was measured by a five-point ordinal scale ranging from 0 to 4 (0 = could not complete the task; 4 = could complete the task independently), making a possible total ranged of 0–56, with higher scores indicating better balance (Downs et al., 2014). During evaluation, researchers evaluated and scored participants by observing their performance.

### 2.3 Tactile sensation tests

The tactile sensitivity of the dominant foot was tested using a set of Semmes-Weinstein monofilaments (North Coast Medical, Inc., Morgan Hill, CA, United States; ICC value, 0.83–0.86) (Collins et al., 2010). The six sizes of monofilaments used herein were 2.83, 3.61, 4.31, 4.56, 5.07, and 6.65, respectively. Pressure was applied until the monofilament formed a C-shaped bend (bend 90°), where the applied force values were 0.07, 0.4, 2, 4, 10, and 300 g. Then, participants were placed in a supine position on the treatment table and randomly tested for tactile sensitivity in the heel, arch, first and fifth metatarsals, and big toe. Thinner monofilaments were used at the beginning of the test and were gradually increased to thicker monofilaments until the participant was able to detect thorough their touch. The sensitivity threshold was determined by the minimum monofilament gauge detected correctly (Ünver and Akbaş, 2018). Better tactile sensitivity was indicated by the lower sensitivity threshold with the lowest threshold detected recorded for data analysis.

### 2.4 Proprioception tests

The proprioceptive thresholds were assessed using a proprioceptive tester, which showed good test-retest reliability (Toshimi, Jinan, Shandong, China; ICC value, 0.737–0.935) (Sun et al., 2015). Proprioceptive thresholds were collected during ankle dorsi/plantarflexion and knee flexion/extension. The apparatus consisted mainly of an operating panel, a steel frame for suspension of the lower limbs, and two movable pedals. At the beginning of the test, the researcher turned on the movable pedal with a button on the control panel. During both ankle and knee proprioceptive tests, the shank was in slight contact with the pedal surface and perpendicular to the pedal surface, while the knee and hip joints were flexed at a 90° angle. Participants were seated in a test chair and wore eye masks and headphones playing music to eliminate potential environmental visual and auditory stimuli while asking the participants to focus their attention. As soon as participants could feel the movement, they were required to press a hand switch to cease the movement of the platform and subsequently identify the direction of the movement. All participants wore socks with uniform material and thickness for the proprioception test. The test was considered successful if participants correctly identified the direction of motion. Three trials were used in each direction for the final data analysis.

### 2.5 Muscle strength tests

Strength testing was assessed using the IsoMed 2000 strength testing system (D. & R. Ferstl GmbH, Hemau, Germany; ICC value, 0.77–0.98) (Gonosova et al., 2018). During the ankle muscle strength test, participants were placed in the supine position with the hip and knee joints in full extension. Ankle motion ranged from 5° of dorsiflexion to 30° of plantarflexion. Meanwhile, in the hip strength test, participants were placed in a lateral position with the hip fully extended, the non-test leg slightly flexed, and the test leg with the knee extended. Additionally, the participant was secured to the bed by a belt around the pelvis and the non-test leg to maintain stability. Moreover, the test leg was stabilized by the belt and the participant was asked to abduct the tested leg with maximum force with a range of motion from 0° to 30°. Participants were then asked to perform a maximum isokinetic force test at an angular velocity of 10°/s and a break was given of at least 2 min between two consecutive trials. Three tests were conducted in each direction.

### 2.6 Statistics

SPSS 26.0 was used to perform the statistical analysis. The Shapiro-Wilk test was then used to check the normality of the data distribution. The means and standard deviations of all outcome variables were descriptively analyzed. Meanwhile, one-way ANOVA (normality) or Kruskal-Wallis H tests (non-normality) were used to compare differences between aging groups. Post hoc analysis was performed using Bonferroni when there were significant differences between groups. Furthermore, Pearson (normality) or Spearman (non-normality) correlations were used to determine the relationship of BBS with tactile sensation, proprioception, and muscle strength in each group. Participants’ heights were also adjusted as covariates. The thresholds of correlation coefficient (r) were as follows: >0.5 (strong); 0.5–0.3 (moderate); 0.3–0.1 (weak); 0.1–0 (trivial) (Cohen, 1988).

## 3 Results

All variables except BBS, tactile sensation, and proprioception were normally distributed. A one-way ANOVA showed significant differences in age (*p* < 0.001) and height (*p* = 0.014) among the three groups. However, no statistical differences were detected in weight and body mass index (BMI) among the three groups (Table 1).

**TABLE 1:** Participants’ information.

Group	YG	MG	OG	*p*
	(*n* = 56, female = 29)	(*n* = 57, female = 28)	(*n* = 53 female = 36)	
Age (years)	65.45 ± 2.15	74.86 ± 3.11	85.21 ± 2.74	**<0.001**
Height (m)	1.64 ± 0.08	1.62 ± 0.07	1.60 ± 0.07	**0.014**
Weight (kg)	65.78 ± 10.94	65.07 ± 9.26	62.81 ± 7.83	0.241
BMI (kg/m^2^)	24.39 ± 3.18	24.72 ± 3.23	24.56 ± 2.73	0.855

Presented as mean ± standard deviation. YG, The younger-aged groups (60–69 years); MG, The middle-aged groups (70–79 years); OG, The older-aged groups (≥80 years); BMI: Body mass index. Bold: *p* < 0.05.

The descriptive statistics of BBS, tactile sensation, proprioception, and muscle strength are shown in Table 2. Results revealed significant differences in BBS scores, tactile sensation, proprioceptive, and muscle strength across the three age groups. A higher score of BBS was observed in the YG compared to the MG (*p* = 0.001) and OG (*p* < 0.001). The MG had worse tactile sensation in the great toe (*p* = 0.035) and heel (*p* = 0.047), had higher proprioception threshold of knee flexion (*p* = 0.030) and extension (*p* = 0.030), and ankle plantarflexion (*p* = 0.010) and dorsiflexion (*p* = 0.039), and had less muscle strength in ankle plantarflexion (*p* = 0.010) and hip abduction (*p* = 0.002) compared to the YG. Meanwhile, the OG had worse tactile sensation in the great toe (*p* = 0.005), higher proprioception threshold of knee flexion (*p* < 0.001) and extension (*p* < 0.001), ankle plantarflexion (*p* < 0.001) and dorsiflexion (*p* < 0.001), and had less muscle strength in ankle plantarflexion (*p* < 0.001) and dorsiflexion (*p* < 0.001), and hip abduction (*p* < 0.001) compared to the YG. They also had higher proprioception threshold of knee flexion (*p* = 0.021) and extension (*p* < 0.001), ankle dorsiflexion (*p* = 0.001), and less muscle strength in ankle plantarflexion (*p* < 0.001) and dorsiflexion (*p* = 0.001), and hip abduction (*p* = 0.010) compared to the MG.

**TABLE 2:** Descriptive characteristics of muscle strength, tactile sensation, and proprioception.

Variables		YG	MG	OG	*p*
BBS		54.93 ± 1.18^a^ ^,^ ^b^	53.46 ± 1.93^c^	50.25 ± 2.66	**<0.001**
Tactile sensation (gauge)	Great toe	4.18 ± 0.53^a^ ^,^ ^b^	4.35 ± 0.56	4.55 ± 0.81	**0.015**
	1st Metatarsal	4.26 ± 0.61	4.24 ± 0.59	4.32 ± 0.48	0.112
	5th Metatarsal	4.36 ± 0.46	4.34 ± 0.48	4.43 ± 0.48	0.386
	Arch	4.41 ± 0.40	4.47 ± 0.64	4.47 ± 0.51	0.492
	Heel	4.51 ± 0.50^a^	4.65 ± 0.57	4.73 ± 0.66	**0.025**
Proprioception (^O^)	Knee flexion	2.25 ± 1.41^a^ ^,^ ^b^	2.91 ± 1.95^c^	3.86 ± 2.35	**<0.001**
	Knee extension	2.46 ± 1.39^a^ ^,^ ^b^	3.33 ± 2.60^c^	4.05 ± 2.62	**<0.001**
	Ankle plantarflexion	1.99 ± 1.05^a^ ^,^ ^b^	3.21 ± 2.59	5.88 ± 4.38	**<0.001**
	Ankle dorsiflexion	2.29 ± 1.76^a^ ^,^ ^b^	3.42 ± 3.18^c^	5.86 ± 4.00	**<0.001**
Strength (N*m/kg)	Ankle plantarflexion	0.46 ± 0.17^a^ ^,^ ^b^	0.35 ± 0.13^c^	0.23 ± 0.13	**<0.001**
	Ankle dorsiflexion	0.25 ± 0.06^b^	0.22 ± 0.07^c^	0.17 ± 0.07	**<0.001**
	Hip abduction	0.51 ± 0.16^a^ ^,^ ^b^	0.41 ± 0.16^c^	0.32 ± 0.15	**<0.001**

Presented as mean ± standard deviation. YG, The younger-aged groups (60–69 years); MG, The middle-aged groups (70–79 years); OG, The older-aged groups (≥80 years); Bold: *p* < 0.05.

^a^ Between-group differences of younger- and medium-older adults.

^b^ Between-group differences of younger- and oldest-older adults.

^c^ Between-group differences of medium- and oldest-older adults.

The correlations of BBS with tactile sensation, proprioception, and muscle strength are shown in Table 3. Among the YG, the BBS was weakly to moderately correlated with proprioception of knee flexion (*r* = −0.351, *p* = 0.009) and extension (*r* = −0.276, *p* = 0.041), and ankle plantarflexion (*r* = −0.283, *p* = 0.036). The BBS was moderately to strongly correlated with muscle strength of ankle plantarflexion (*r* = 0.326, *p* = 0.015) and hip abduction (*r* = 0.415, *p* = 0.002). However, none of the BBS was correlated with tactile sensation test results. Among the MG, the BBS was weakly correlated with proprioception of ankle plantarflexion (*r* = −0.291, *p* = 0.030) and was moderately correlated with muscle strength of ankle plantarflexion (*r* = 0.448, *p* = 0.001) and dorsiflexion (*r* = 0.356, *p* = 0.007). However, none of the BBS was correlated with tactile sensation test results. Among the OG, the BBS was weakly to moderately correlated with muscle strength of ankle plantarflexion (*r* = 0.276, *p* = 0.034) and hip abduction (*r* = 0.324, *p* = 0.015), with none of the BBS still being correlated with tactile sensation and proprioception test results.

**TABLE 3:** The age-specific correlations of BBS with muscle strength, tactile sensation, and proprioception.

Variables		BBS					
		YG		MG		OG	
		*r*	*p*	*r*	*p*	*r*	*p*
Tactile sensation (gauge)	Great toe	−0.101	0.461	−0.213	0.115	−0.264	0.051
	1st Metatarsal	0.047	0.732	−0.117	0.391	−0.016	0.909
	5th Metatarsal	0.067	0.625	−0.169	0.209	0.089	0.513
	Arch	−0.045	0.745	−0.256	0.056	0.062	0.648
	Heel	−0.129	0.347	−0.021	0.878	−0.004	0.975
Proprioception (^O^)	Knee flexion	−0.351	**0.009**	−0.241	0.073	−0.126	0.354
	Knee extension	−0.276	**0.041**	−0.060	0.662	−0.101	0.460
	Ankle plantarflexion	−0.283	**0.036**	−0.291	**0.030**	−0.134	0.324
	Ankle dorsiflexion	−0.196	0.151	−0.207	0.125	−0.192	0.257
Strength (N*m/kg)	Ankle plantarflexion	0.326	**0.015**	0.448	**0.001**	0.276	**0.039**
	Ankle dorsiflexion	0.126	0.360	0.356	**0.007**	0.096	0.480
	Hip abduction	0.415	**0.002**	0.244	0.071	0.324	**0.015**

YG, The younger-aged groups (60–69 years); MG, The middle-aged groups (70–79 years); OG, The older-aged groups (≥80 years); *r*: correlation coefficient. Bold: *p* < 05. Adjusted for height.

## 4 Discussion

This study compared balance control and its potential contributors, tactile sensation, proprioception, and muscle strength and investigated the relationship of balance control with its three potential contributors among older adults of different ages. Results partly support our hypotheses: there were significant differences observed in balance control, tactile sensation, proprioception, and muscle strength across the three groups. The balance control was correlated with proprioception and muscle strength in the YG and MG, but it was only correlated with strength in the OG. None of the tactile sensation was correlated with balance control in the three groups.

A lower score of BBS was observed in the OG compared to the other two groups. The findings were consistent with previous studies that a decline in the balance functions occurs with age (Cuevas-Trisan, 2017; Thomas et al., 2019). Therefore, the balance function is essential to prevent falling in older adults. Changes in the central nervous system and neuromuscular system properties with age may negatively affect the performance of balance control among older adults (Gschwind et al., 2013).

The outcomes have shown that the OG had worse tactile sensation compared to the MG and YG, and no correlations were confirmed between tactile sensation and balance control among the three age groups. Our findings are consistent with those of some previous reports (Shaffer and Harrison, 2007). Aging may affect the mechanical properties of the skin as well as changes in skin receptor density, morphology, and physiology, likely leading to a reduced tactile sensation among older adults (Peters et al., 2016). The great toe and heel are located at the front and rear of the foot and at the most distal part of the body, while the tactile sensation deteriorates in a distal to proximal manner (Toledo and Barela, 2014). This may explain the significant group differences at both the great toe and heel. Further, the tactile sensation was not correlated with balance control, which has been documented in previous studies (Ünver and Akbaş, 2018). However, some studies reported that tactile sensation was correlated with balance control (Bretan et al., 2010). This conflict may be explained by the difference in monofilament specifications used in the different experiments. Bretan et al. (2010) used only one monofilament sized 5.07 (10 g), while six monofilament sizes were used to test tactile sensation in the current study which allowed for more comprehensive data. By using six monofilaments of different sizes, tactile sensation threshold of the older adult could be measured more accurately. Moreover, the lack of correlation between tactile sensation and balance control among older adults may also be due to the compensation of other sensory information for tactile sensation when maintaining balance such as proprioceptive, visual, and vestibular sensory information (Chen et al., 2012; Ferlinc et al., 2019).

Results showed that OG had a higher proprioception threshold compared to the other two groups, and proprioception was related to balance control in YG and MG, but not in the OG. Proprioception therefore declines with age, which has been well documented in existing studies (Henry and Baudry, 2019). The decline of proprioception may be related to age-related alterations in the musculus and its neural pathways, which may lead to deficits in the processing and input of proprioceptive signals (Ferlinc et al., 2019). It may also be associated with muscle dysfunction and degeneration of articular cartilage (Wingert et al., 2014). Here, joint motion sense was used to represent proprioception, rather than joint position sense. Notably, the participants’ performance on joint position sense was overall more erratic compared with joint motion sense (error varied more among trials) (Reider et al., 2003). Joint position sense is a more complex test than joint motion sense, which requires coordinated afferent and supraspinal efferent output. In joint motion sense, subjects only have to signal when motion is sensed. In joint position sense however, the subjects must try to remember a position and then accurately reproduce it. Thus, there may have been more of a study effect in joint position sense findings with the older adults who were tested on multiple occasions. They may have tended to concentrate more than the external controls and may also have benefited from a learning effect (Reider et al., 2003). Moreover, joint force sensing was not used because it did not show good reliability (Benjaminse et al., 2009) due to the difficulty for participants to maintain a uniform test posture during the test and because participants may use different strategies to complete the test (Benjaminse et al., 2009). There was no significant difference in the proprioception of ankle plantarflexion between the MG and OG groups. This could be due to the larger standard deviation in the OG group. Sensation deteriorates in a distal to proximal manner (Toledo and Barela, 2014), and more distal muscles, such as the soleus, which is one of the ankle plantarflexors, may deteriorate more than other muscles and enlarge the variance in plantarflexion proprioception in the OG group. Knee proprioception was not correlated with BBS scores in the MG and OG groups, which may be due to the ankle or hip strategies being often used to return the body to balance when disturbed instead of the knee strategy (Horak, 2006). The proprioceptive feedback in the ankle joint may also be more important in regulating muscle activity compared to the knee joint (Mayer et al., 2018). Our findings indicated that dorsiflexion proprioception is not correlated with BBS scores, while plantarflexion proprioception is correlated with BBS scores. This may be because individuals rely more on the contraction of the plantarflexor (rather than the dorsiflexor) to complete most of the BBS tasks, such as from sitting to standing or when turning. Previous studies coincide with this and point out that ankle proprioception is crucial for balance and relies upon accurate input from plantarflexor, such as the calf triceps (Reynolds et al., 2020). Meanwhile, proprioception was correlated with balance control in both YG and MG groups, but not among the OG. Proprioception is the sensory system that provides information about motor activity levels to the central nervous system, and its inputs are essential for balance control (Horak, 2006). However, the decline in proprioception may significantly increase body sway and affect mobility, thereby resulting in impaired balance and a higher possibility of falling (Ferlinc et al., 2019). Therefore, proprioception was related to balance control in the YG and MG groups—older adults in these two groups could thus rely on proprioception to control balance. However, proprioception was not related to balance control in OG, which may be that the proprioception among the OG could not provide sufficient information on balance control. This inferred the deterioration of proprioception (to a certain extent) among older adults over 80 years, and did not continue to provide any meaningful information on balance control in older adults. Furthermore, proprioception deterioration may have a greater effect on balance control among older adults older than 80, which may also plausibly account for the increased risk of falling of those in the OG.

Results herein have shown that weaker muscle strength was observed in the OG compared to the other two groups, and that muscle strength was related to balance control among the three age groups. This coincides with the outcomes of other studies where aging is associated with a significant decline in muscle strength (Doherty, 2001). Additionally, balance control was correlated with muscle strength among older adults, which is also consistent with previous studies (Tavakkoli et al., 2021). Similarly, Tavakkoli et al. (2021) found a significant correlation between muscle strength of ankle plantarflexion with balance among older adults. These observations also contrast those of other studies. Muehlbauer et al. (2012) found no significant correlation between muscle strength and balance among older adults. The inconsistent results may be because their study measured muscle strength during isometric contractions, whereas the current study measured muscle strength during isokinetic contractions (Muehlbauer et al., 2012). Isokinetic contractions involve dynamic muscle contraction and can better reflect dynamic balance control, compared to isometric contractions which involve static contractions (Song et al., 2021). Although muscle strength decreases with age, the relationship between muscle strength with balance control in all age groups may indicate that muscle strength still plays an important role in maintaining balance control among older adults.

The following limitations are seen herein: first, only the effects of tactile sensation, proprioception, and muscle strength on balance control were investigated in the current study. Nevertheless, other factors might also affect balance control among older adults such as visual, cognitive functions, or vestibular factors. Second, all the participants were recruited from the same city and had similar backgrounds. Therefore, it is recommended that future studies should include populations with different characteristics.

## 5 Conclusion

Senior older adults aged over 80 have worse balance control, deteriorated tactile sensation and proprioception, and less muscle strength compared to their younger counterparts. Proprioception and muscle strength were correlated with balance control in the 60–69 and 70–79 age groups, while only strength, but not proprioception, was correlated with balance control among older adults over 80. The worsened proprioception among older adults aged over 80 years could be the key for their increased falling.

## Data Availability

The datasets presented in this study can be found in online repositories. The names of the repository/repositories and accession number(s) can be found below: http://doi.org/10.57760/sciencedb.03500.
